# Glyco-gold nanoparticles: synthesis and applications

**DOI:** 10.3762/bjoc.13.100

**Published:** 2017-05-24

**Authors:** Federica Compostella, Olimpia Pitirollo, Alessandro Silvestri, Laura Polito

**Affiliations:** 1 Department of Medical Biotechnology and Translational Medicine, University of Milan, Via Saldini 50, 20133 Milan, Italy; 2 Department of Chemistry, University of Milan, Via C. Golgi 19, 20133 Milan, Italy,; 3 CNR – ISTM, Nanotechnology Lab., Via G. Fantoli 16/15, 20138 Milan, Italy

**Keywords:** gold nanoparticles, glyco nanoparticles, glyconanomaterials, immunology, targeting

## Abstract

Glyco-gold nanoparticles combine in a single entity the peculiar properties of gold nanoparticles with the biological activity of carbohydrates. The result is an exciting nanosystem, able to mimic the natural multivalent presentation of saccharide moieties and to exploit the peculiar optical properties of the metallic core. In this review, we present recent advances on glyco-gold nanoparticle applications in different biological fields, highlighting the key parameters which inspire the glyco nanoparticle design.

## Introduction

Carbohydrates are well recognized as crucial biomolecules for their capability to produce an enormous number of biocodes which are translated into their ability to promote or suppress a plethora of biological events [1]. Nevertheless, carbohydrates interact with their ligands in a very weak manner, so much faint that nature found in the multi-presentation (the so called “glycocalix”) a solution to have efficient carbohydrate interactions. In fact, the sum of binding affinities for single and isolated interaction is ever lower than those measured when a glyco-multivalent presentation is exploited [2]. Following nature’s design, carbohydrate multivalent systems are, at the present time, the most common strategy used to study weak carbohydrate–carbohydrate or carbohydrate–protein interactions and the resulting biological processes [3–4].

The increasing ability to manipulate material at the nanosize allowed the development of many glyco nanoparticles helpful in a wide range of applications, from the drug delivery to the imaging [5–7]. Among the large number of nanomaterials, gold is one of the main exploited scaffolds for producing glyco nanoparticles [3–4]. First of all, gold is an extremely inert and biocompatible material. At nanosize, gold nanoparticles (AuNPs) are characterized by unique optical features which result extremely useful in many diagnosis and affinity studies or protocols. Moreover, gold can be easily and covalently decorated on its surface by exploiting the strong soft–soft interaction between a Au atom and sulfur [8]. Therefore, glyco-gold nanoparticles (GAuNPs) represent a smart, multimodal and versatile nanoplatform to develop carbohydrate-based nanotechnology and nanomedicine systems.

The present review focuses its attention only toward the synthesis of gold-based nanoparticles coated with carbohydrates. A first introduction related to the main protocols to produce glyco-gold nanoparticles is followed by a section where the main parameters which should be taken into account for designing the best performing nanosystem are discussed. Then, three sections summarize the achievements and ongoing researches published in the last four years, from 2013 to 2016 with a special focus on glyco-gold nanoparticle applications (in biosensing field, as drug carriers or contrast agents and as effective tool in the immunological field).

## Review

### Synthesis of glyco-gold nanoparticles

**Figure 1:**
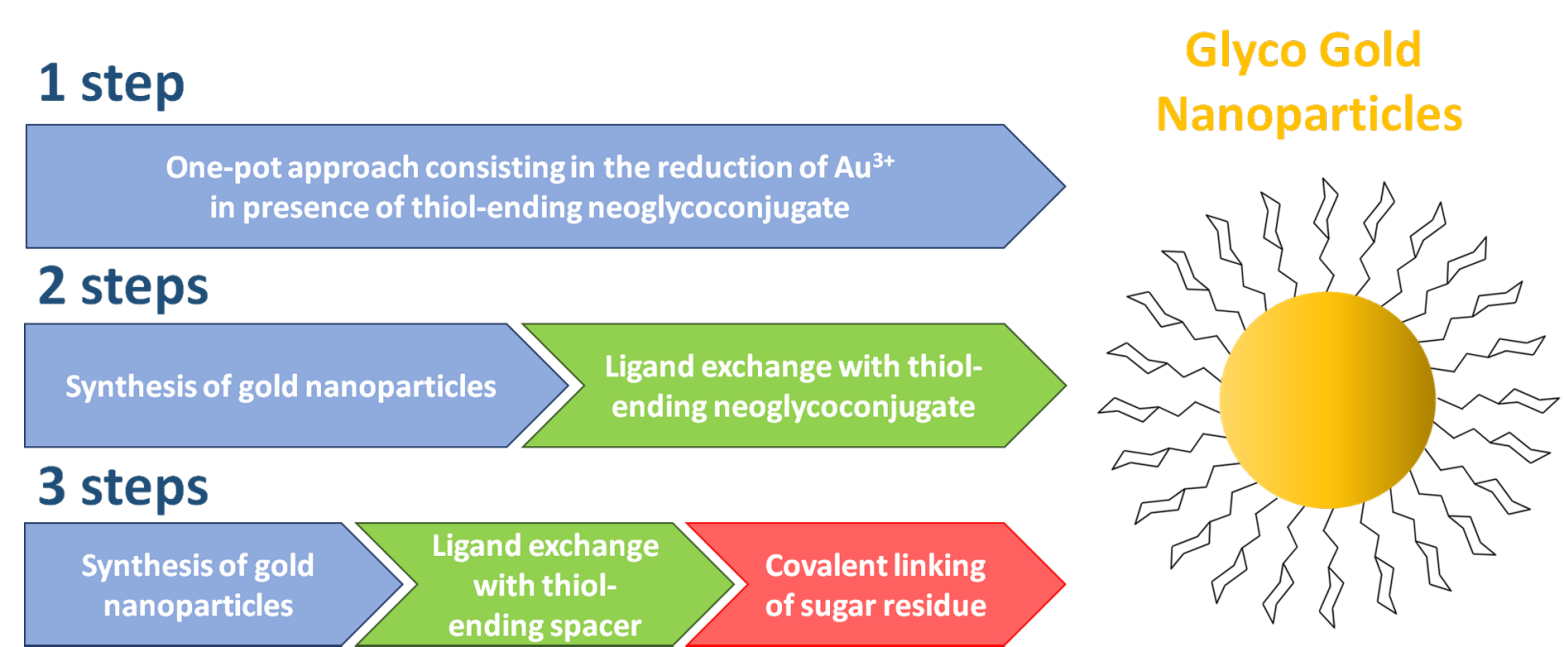
Schematic overview on the glyco-gold nanoparticles synthetic approaches.

Despite the great inertness of this noble metal, Au can form stable bonds with sulfur-containing compounds (i.e., thiols or disulfides) [9–10] and it is possible to easily and robustly functionalize AuNPs with organic molecules. The synthetic approaches to GAuNPs can be classified into three major categories (Figure 1).

The first one consists in a one-step preparation of ultrasmall spherical gold nanoparticles coated with a thio-glucoside [4,11–16]. The process, developed in 2001 by Penadès and co-workers [15] is a modified Brust–Shiffrin method [17], based on the in situ Au^3+^ reduction by means of NaBH_4_ in the presence of thiol-ending neoglycoconjugates. Following this approach, a rapid synthesis of AuNPs characterized by elevated glycan density on the NP surface and a 1–10 nm diameter range can be obtained [4]. Moreover, this synthetic procedure allows to introduce different types of carbohydrates and other ligands (i.e., polyethylene chains, lipids, peptides, DNA, RNA or fluorescent dyes) in controlled ratios [4]. A modification of this technique consists in the application of the “thiol for thiol” ligand exchange method, which allows the superficial diversification by introducing small amounts of different ligands after the AuNP synthesis [11–12,18]. Recently, the Seeberger group reported a straightforward one-pot method to prepare glucose-stabilized ultrasmall AuNPs, by simply mixing at room temperature Au^3+^ salts and thio-glucoside as reducing and stabilizing agents, without the addition of NaBH_4_ [19].

The two-step synthetic procedure consists in the initial synthesis of AuNPs stabilized by temporary compounds (i.e., citrates, amines or phosphines), followed by the ligand exchange protocol to introduce the thiolated saccharide molecules [20–22]. In this frame, the Turkevich method [23] is the most common protocol to produce citrate-capped spherical nanoparticles, with diameter size between 10 and 50 nm. Noteworthy, by employing surfactant or templating agents (i.e, AgNO_3_ or cetyltrimethylammonium bromide, CTAB), a control over the core morphology can be implemented with this methodology, as reported for the synthesis of mannose and galactose functionalized nanorods and nanostars [24] or glucosamine-coated nanostar AuNPs [22]. Respect to other methodologies, the main drawbacks of this protocol are the longer reaction times (12–24 hours), the lower glycan loading and the difficulty for a fine control of the organic shell composition.

The last category collects all the protocols based on chemical conjugations of sugar residues to ligands displayed on the metal surface of pre-formed AuNPs. Despite the longer times required for the NP synthesis and the first coating, the main advantages of this approach are the use of a lower amount of precious sugar residues and the possibility to exploit different orthogonal surface functionalizations. Reactions employed for this purpose (i.e., click reaction, amidation, conjugation via carbonyldiimidazol and perfluorophenyl azide (PFPA) photo-coupling) have to be compatible with water, the common medium for AuNP preparation [25–29]. One example of the three-step approach was described by Tian and co-workers [28]. AuNPs were firstly coated by a monolayer of dithiol-cyclooctyne spacer. Then, the alkyne moiety reacted with mannosyl azide through a copper-free strain-promoted alkyne-azide cycloaddition (SPAAC), affording the GAuNPs. Alternatively, Yan and co-workers developed a three-step photocoupling procedure for the immobilization of carbohydrates on the nanoparticles surface [27]. The AuNPs were firstly coated with a PFPA-thiol monolayer via a ligand-exchange reaction, then carbohydrates were immobilized by means of a photocoupling reaction.

### Glyco-gold nanoparticles: design and structural properties

**Figure 2:**
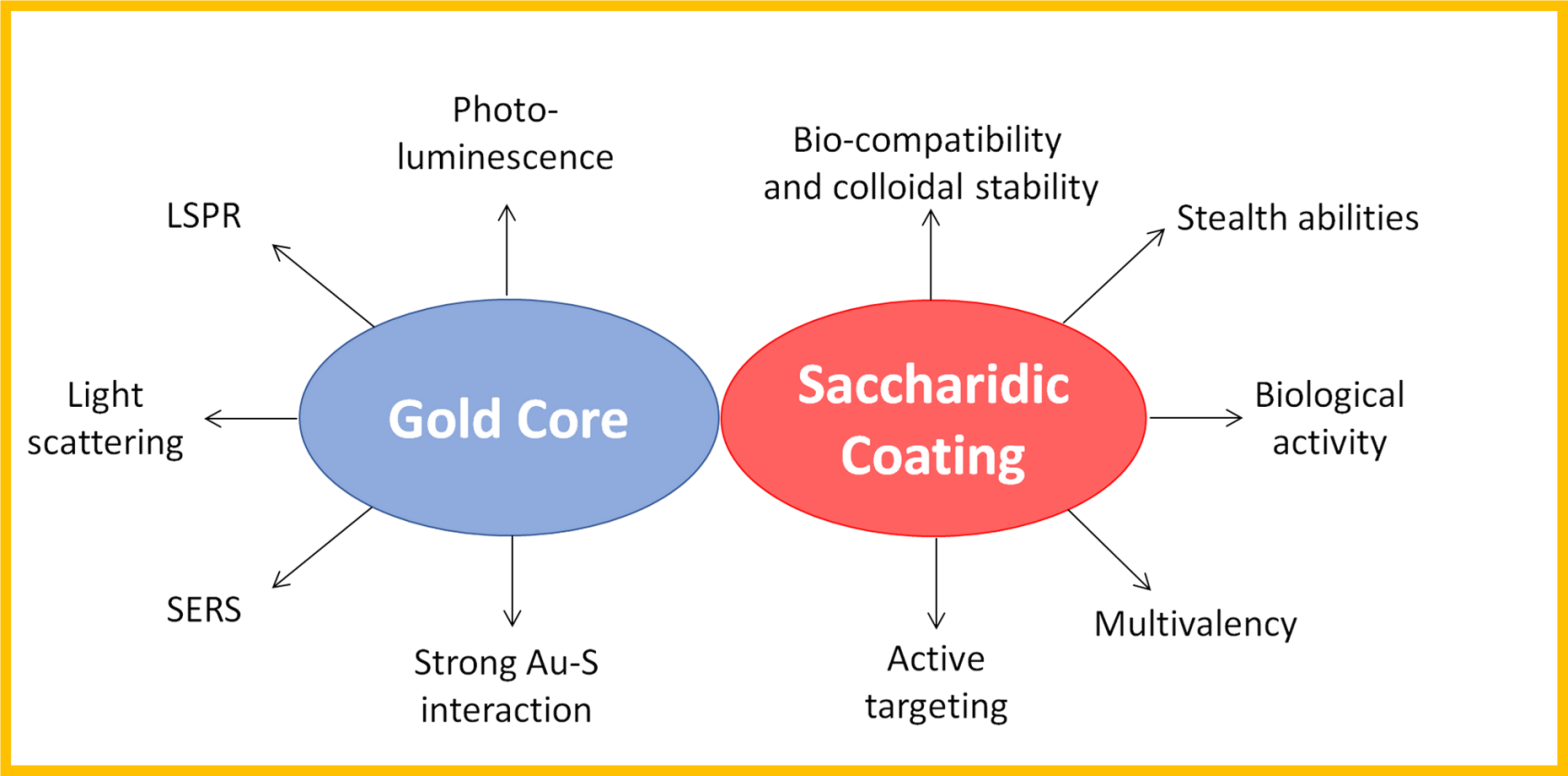
Glyco-gold nanoparticles: metallic core and glyco-coating contribute to the production of the versatile glyco-nanomaterials.

GAuNPs are constituted by two main entities, the metallic core and the organic surface (i.e., mono- oligo- and polysaccharides). The features of these entities are tightly related to their application and effectiveness (Figure 2). Therefore, a careful design of GAuNP is required to obtain the best performing system.

#### Gold core: optical properties

AuNPs, widely employed for biomedical applications, are characterized by inert nature, resisting to air oxidation and corrosion [30]. This chemical non-reactivity and inertness make AuNPs an outstanding candidate for the development of in vitro and in vivo devices [31–32]. Furthermore, in vitro and in vivo short term reduced toxicity of AuNPs has been widely documented [10,33–38]. AuNPs own a number of peculiar optical properties, strongly dependent on the size and the morphology of the metallic core. When AuNP dimension is comparable to the gold Fermi wavelength, the nanoparticles acquire photoluminescence properties [31]. These small clusters typically present a diameter between 0.5 and 2 nm. When clusters are bigger, they do not present fluorescent emission but they show a localized surface plasmon resonance (LSPR) around 520 nm. This phenomenon consists in a collective oscillation of the free electrons of the metal, induced by the light impinging on the AuNPs [31,39]. The AuNP surface plasmon resonance is dependent upon a vast number of parameters like size, shape, morphology and environment surrounding AuNPs [40]. By controlling these parameters, it is possible to tune the LSPR peak through the visible and near IR spectra. As effect of the LSPR process, the incident light is strongly attenuated and intense electric fields are generated in the proximity of the AuNP surface. These electric fields are of great interest because they can be employed to enhance the intensity of Raman signals close to the NP surface, widely explored in biomedical diagnostics (surface enhanced Raman spectroscopy, SERS). This effect is even more intense when the nanoparticles possess an irregular shape, generating an anisotropic distribution of the magnetic field [41]. AuNP optical properties have been widely exploited in the design of GAuNPs with interesting applications such as the colorimetric biosensors. Based on the LSPR phenomenon, the controlled aggregation and de-assembling of AuNPs is reflected in a detectable variation of the colloidal solution color. Therefore, several biosensors for the detection of proteins [42], lectin [25,27,43], cancer biomarkers [28] and viruses [44–45] were developed. Photoluminescence properties of ultrasmall gold nanoclusters and nanodots have been exploited to detect *Escherichia coli* (a bacteria containing mannose-binding receptors) [46] and thyroglobulin in serum [47] and to study the internalization pathway in dendritic cells, employing confocal laser microscopy (CLSM) and flow cytometry [48]. Moreover, even techniques based on light scattering, like dark field microscopy (DFM), resulted convenient for the biosensing application, considering that the light scattered by a single AuNP can be about 1 million times more intense than the fluorescence emitted by a dye [49–50].

#### Gold core: the size and the shape role

AuNP core dimension and shape are important parameters to take into consideration for the multivalent carbohydrate presentation [51–53]. In fact, a denser ligand packing can be obtained when NP dimensions are bigger and the curvature radius is reduced. This observation was already highlighted by Lin et al., during their studies on the interactions among Shiga toxins and multivalent GAuNPs [52]. The authors, comparing the affinities of 4, 13 and 20 nm AuNPs, demonstrated that, when the diameter grows and the surface becomes flatter, the interaction with the proteins binding sites is facilitated. Besides the AuNP size, the NP shape can affect the effectiveness of active molecules displayed on the metallic surface. In particular, an in vitro study performed by Mitragotri and co-workers reported that the endothelial targeting specificity could be enhanced by modulating the NP shape. By using a microfluidic synthetic vascular network the authors demonstrated that rod-shaped NPs exhibit elevated specific accumulation in comparison to spherical ones [54]. Recently, Kikkeri et al. published two interesting papers evaluating the impact of the AuNP shape on the carbohydrate presentation and activity [24,55]. In the first work the authors reported the employment of GAuNPs for the bacterial recognition and bacterial infection inhibition. Three different morphologies of AuNPs (sphere, rod and star-like NPs) coated with galactose and mannose derivatives were employed for the quantification of their binding affinity to *E. coli*, suggesting that each shape could induce a different bacterial adhesion. Indeed, gold nanorods presented a bacteria detection limit of 0.03 ± 0.01 µg/mL, 80-folds more sensitive than spherical and star shaped AuNPs. This discrepancy has been ascribed to the difference in the relative amount of mannose involved in the NP-bacteria interaction. The same group in 2016 investigated as well the effect of the AuNP morphology on carbohydrate–protein interactions in mammalian cells. In particular, the authors studied the shape-dependent uptake of mannose- and galactose-functionalized AuNPs in different cancer cells. By employing an enzyme-linked immunosorbent assay (ELISA), the selectivity and sensitivity of the binding were demonstrated to be dependent on the sugar nature and on the NP morphology. The cellular uptake experiments on HeLa, HepG2 and MDA-MB-231 cells showed in fact an higher clathrin-mediated internalization for rod-shaped AuNPs in comparison to spherical and star-shaped AuNPs.

#### Saccharidic functionalization: the coating density

Besides the inorganic core, the surrounding organic shell plays a crucial role in GAuNP chemical-physical properties. The organic functionalization, in fact, impart the stealth ability and biocompatibility of the nanomaterials. Moreover, when the metallic surface is decorated with an active target, the GAuNPs can act as a smart probe, even exploiting the multivalent effect.

Several studies were focused on the investigation of synthetic techniques to improve saccharide density on the nanomaterial surface [45,56–58]. Prosperi and co-workers coated dodecanthiol AuNPs with manno-calixarenes exploiting hydrophobic interactions, obtaining an efficient targeting against cancer cells [56]. Similarly, a reversible addition−fragmentation chain transfer (RAFT) polymerization approach has been exploited for the preparation of “multicopy–multivalent” AuNPs, decorated with complex glycan structures. The high degree of ramification resulted in a strong selective recognition [57–58] with the possibility to modulate the glycosylic loading. Even if the carbohydrate loading is important, not always a high carbohydrate density is reflected in an improved affinity and it depends on the biological event observed [18,59–64]. For example, in the case of carbohydrate−protein interactions, the carbohydrate disposition on the NP surface is more important than the loading of the active molecule. Proteins, like lectins, are characterized by different subunits possessing characteristic distances between the active pockets, so a controlled carbohydrate density can result in a better presentation of the molecules [60].

#### Saccharidic functionalization: the importance of the linker

The linker employed to space glycans from the Au surface is another fundamental factor to take into consideration in GAuNP design. Generally, the majority of the spacers are thiol-linkers, exploiting the strong soft–soft interaction between gold and sulfur. The spacer has the crucial role to stabilize GAuNPs in the surrounding media, which is fundamental in the case of colorimetric bioassays where the NP colloidal stability can strongly influence the state of aggregation and, consequently, the analysis results [43,65–66]. Woods et al., in a recent paper, performed a computational study to evaluate the role of the spacer in the glycan–protein interaction [67]. The authors considered different types of spacer in terms of chemical structure, length and rigidity and demonstrated that longer linkers resulted as the best performing ones, giving a better access to the protein pocket. Furthermore, they showed that rigid spacers could hinder the binding, by creating unfavorable interactions with the protein surface, while flexible spacers resulted in a better carbohydrate presentation and subsequent interaction with the target. Schlecht and co-workers experimentally demonstrated that the binding selectivity of GAuNPs toward P-selectin was affected not only by the spacer length but also by the presence of amide bonds next to the protein pocket [51].

#### Saccharidic functionalization: colloidal and stealth ability

GAuNP colloidal stability and stealth abilities in biological media and in intracellular environment is a fundamental issue for the efficacy of these nanomaterials [68]. The use of stealth coatings is an effective strategy to decrease or inhibit the non-specific interaction with plasma proteins (the so called “protein corona”), that can modify GAuNP biological identity, affecting aggregation, and cellular uptake [69–71]. Moya and co-workers deeply studied the intracellular dynamics and aggregation of glucose-AuNPs by employing fluorescence correlation spectroscopy (FCS) [12]. They demonstrated that GAuNPs were ubiquitous distributed inside the cell as single NP or small aggregates, suggesting a strong intracellular stability. Liz-Marzan et al. assessed the ability of glycan ligands to reduce the protein corona formation around rod-shaped AuNPs [71]. Lactose- and polyethylene glycol (PEG)-functionalized gold nanorods demonstrated similar ability in reducing the interaction with proteins, when compared to citrate-AuNPs. Moreover, comparing the macrophage NP uptake, the authors demonstrated that the lactose shell can prevent phagocytosis more efficiently than PEG coating.

A deep knowledge of any aspect which can influence the GAuNP behavior is important to drive the correct design of the nanosystem and is strongly related to the final bioapplication.

### Glyco-gold nanoparticles as nanosensors

GAuNPs are able to create supramolecular networks by interacting with proteins, enzymes or other carbohydrates and this interaction is reflected in a measurable shifting of the LSPR band, due to the aggregation of the metallic NPs. Carbohydrate–carbohydrate interactions (CCIs), generally characterized by *K*_D_ values in the millimolar range, are very difficult to detect. Updated data confirm the crucial role of these bindings in the regulation of many cellular processes [72], however, researches related to the use of carbohydrate-coated gold nanoparticles to investigate CCIs, are still in their infancy and will be not detailed in the present review [73–74]. On the contrary, a vast number of papers reported the use of glyco-gold nanoparticles as smart nanomaterial, able to recognize and interact with proteins, viruses and peptide hormones.

#### Detection of carbohydrate–protein interactions

**Figure 3:**
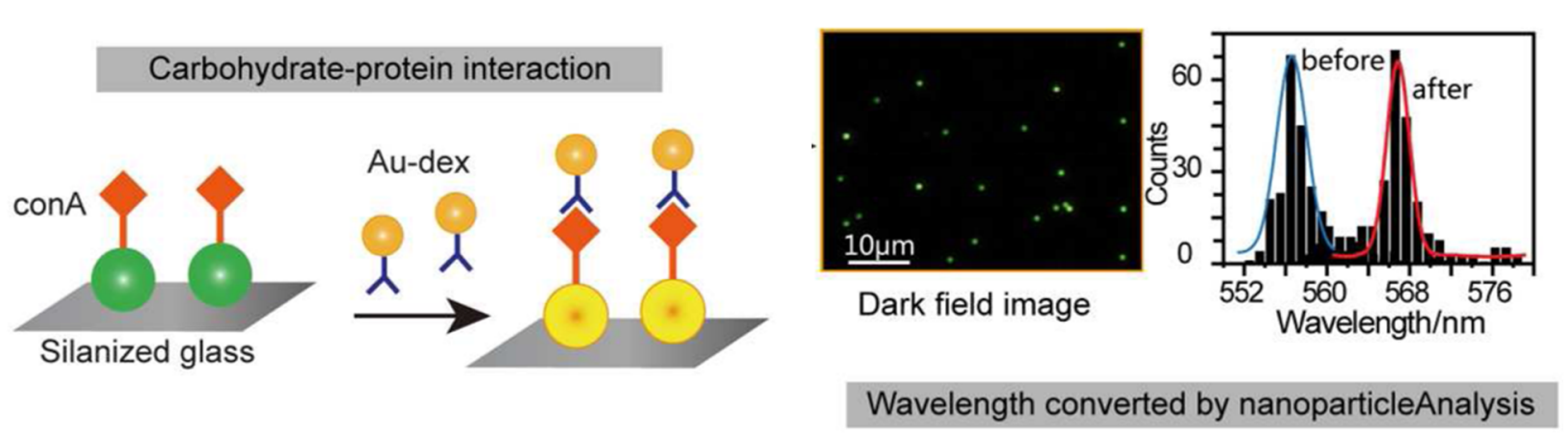
The protein–carbohydrate interactions analyzed by conventional dark field microscopy. The interaction among ConA-functionalized AuNPs (60 nm) and dextran-coated AuNPs (20 nm) are detected by DFM. Reprinted with permission from [50]. Copyright 2015 American Chemical Society.

Carbohydrate–protein interactions have a crucial role in many pathological and physiological cellular functions, acting as a main process correlated with a large number of biological events, such as inflammations, cancer metastasis, fertility, etc. [75–77]. Lectins are the most diffused class of proteins which recognize carbohydrate ligands and they are widely studied to develop systems able to detect lectin–carbohydrate interactions. Yan et al. exploited the use of gold nanoparticles to recognize and differentiate lectins by capping gold surface with eleven different carbohydrate ligands [78]. To achieve their goal, the authors treated GAuNPs with lectins and used the linear discriminant analysis (LDA) to analyze the LSPR shifts and recognize the lectins in a complex sample. A similar approach, based on LDA, has been employed by Gibson et al. to provide discrimination among legume lectins [79]. In their paper, the authors synthesized eleven GAuNPs, by tuning a heterogeneous coatings using only two 2-amino-2-deoxysugars (galactosamine and mannosamine). By means of this approach, and limiting the carbohydrate synthetic efforts, the GAuNPs showed an incremented lectin identification power, at low protein concentrations, enabling an easy tool for sugar–lectin recognition. The glycan–protein interaction has been studied as well employing surface-enhanced Raman spectroscopy (SERS) exploiting AuNP ability in amplifying Raman signals [80]. The greatest advantage in using SERS is the possibility to provide unique spectral signatures describing glycan–protein interactions. Boons and co-workers prepared label-free microarrays of lactose-coated AuNPs (diameter of 60 nm) to study lactose galectin (i.e., galectine 1 and galectin 3) and influenza hemagglutinin interactions. The resulting NPs were deposited and dried on a gold layer and SERS measurements performed. Finally, partial least squares discrimination analysis (PLS-DA) was performed to analyze the SERS spectra, differentiating them in different classes with statistical relevance. Long et al. [50] developed a simple procedure to analyze in real-time the carbohydrate–protein interaction at the single NP level, using for the first time an unusual technique such as the dark field microscopy, DFM (Figure 3). Briefly, the method is based on the interaction among 60 nm AuNPs capped with the protein concanavalin A (ConA, physically adsorbed on the surface) and smaller dextran-coated AuNPs. ConA-AuNPs, immobilized on a glass slide, were treated with dextran-AuNPs and when the two particles interacted, a coupling of the plasmonic oscillations was detected, resulting in a shift of the statistic peak wavelength distribution. The statistical analysis of the peak wavelength was performed starting from the RGB (red, green and blue) information obtained by DFM by analyzing them with a self-developed statistical program (nanoparticleAnalysis).

**Figure 4:**
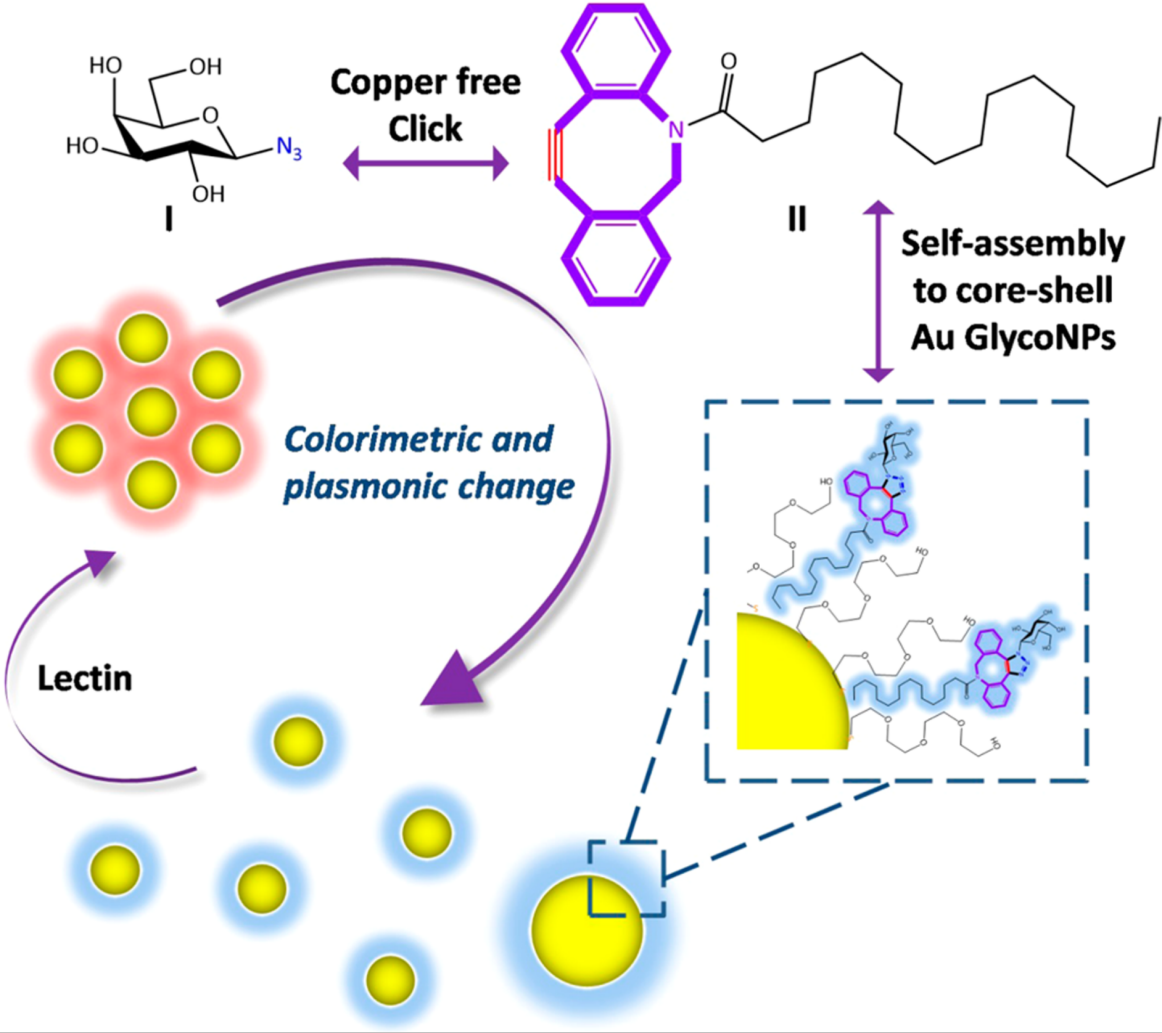
Copper-free cycloaddition (SPAAC) of azido galactoside on cyclooctyne and schematic depiction of Au surface coating. When galactoside-coated AuNPs interact with lectin, AuNPs aggregates, inducing a plasmonic band shift. Reprinted with permission from [25]. Copyright 2015 American Chemical Society.

A very interesting result reports on the design of highly stable carbohydrate-coated gold nanoparticles, able to recognize at subnanomolar and low picomolar concentrations level of ConA, wheat germ agglutinin and *Ricinus communis* agglutinin [43]. In this case, a careful decoration of the gold surface has been carried out by employing a thio-amphiphilic linker to impart improved solubility and flexibility to the glycosyl-ligand. UV–vis spectroscopy and dynamic light scattering measurements have been exploited to detect at low picomolar concentrations lactose-AuNP, mannose-AuNP and GlcNAc-AuNP interactions with their cognate lectins. Moreover, by using an immobilized antibody microarray, it was possible to design a highly sensitive lectin detection, enhanced by silver addition, by easy naked eye. Plasmon resonance spectroscopy and naked eye have been used by Long et al. (Figure 4) to study the interaction of galactoside-AuNPs with peanut agglutinin [25]. In this case, the glycosyl residue is not coated on the gold surface by means of a thiol moiety but exploiting the strain-promoted azide–alkyne cycloaddition (SPAAC) to covalently link an azido galactoside on a lipid cyclooctyne. In this manner, the amphiphilic glyco-lipid can be embedded on PEGylated-gold nanoparticles and the sugar moiety displayed on the outer shell of the nanosystem is available for interaction with lectin.

**Figure 5:**
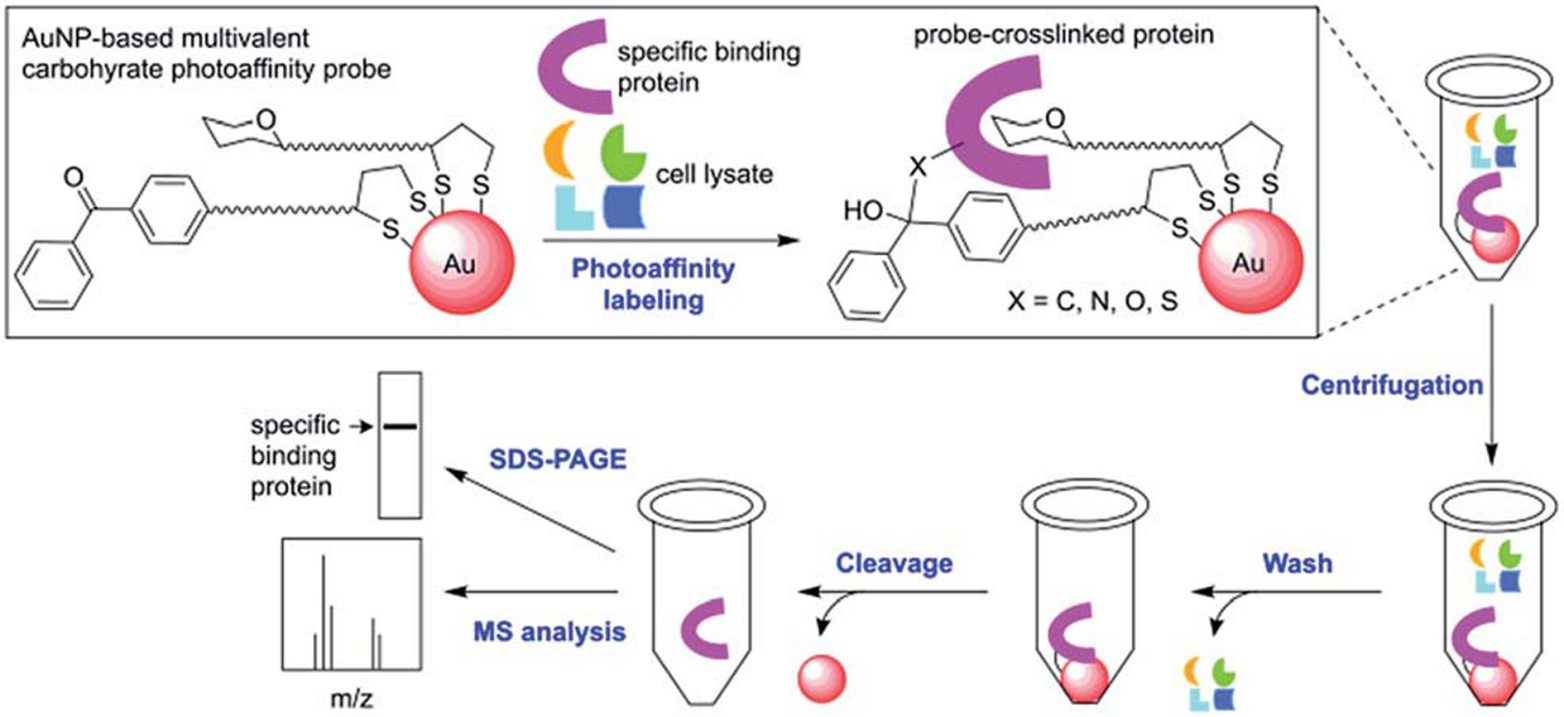
A photoaffinity labeling (PAL) approach based on glyco-gold nanoparticles is able to recognize and isolate only the desired protein. After washing and cleavage steps, the protein can be fully analyzed. Reprinted from [81]. Published by The Royal Society of Chemistry.

In 2016, Sakurai and co-workers [81] combined the multiple display of the carbohydrate moiety on AuNPs with the photoaffinity labeling (PAL), to concentrate and purify proteins by covalent crosslinking (Figure 5).

GAuNPs were synthesized by coating the metal surface via thio-chemistry with a β-D-lactose residue (recognized by a series of lectins, i.e., PNA or *Ricinus communis* agglutinin) and a benzophenone moiety as photoreactive group. GAuNPs were treated with PNA which cross-linked with the photoreactive group, affording a PNA-AuNPs complex. The latter was easily purified by the non-interacting protein via centrifugation and, eventually, the protein was quantified after an appropriate cleavage with 2-mercaptoethanol.

**Figure 6:**
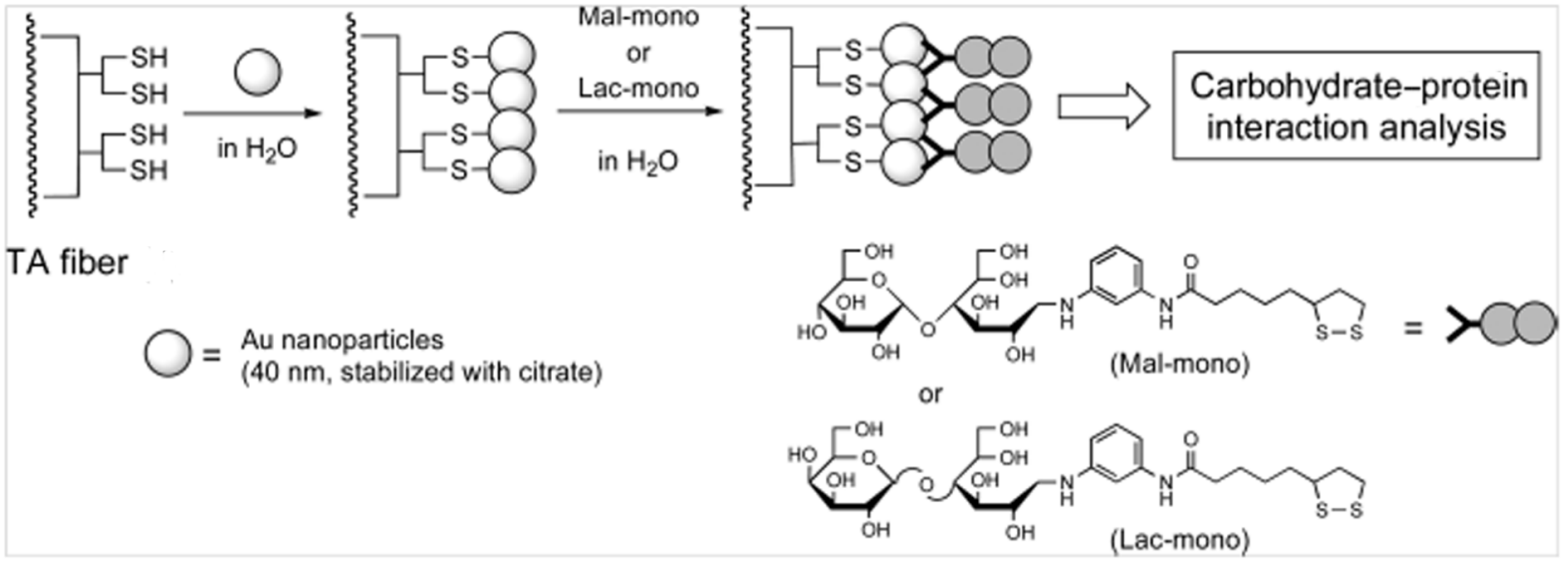
Design of the fiber-type “Sugar Chips”. AuNPs are firstly immobilized on an optical fiber functionalized with α-lipoic acid (TA fiber). Successively, glycosil-thioctic residues were grafted on AuNPs, forming the “Sugar Chips”. Reprinted with permission from [82]. Copyright 2017 American Chemical Society.

A very recent and interesting application showed for the first time the immobilization of AuNPs on the endface of an optical fiber by means of thio-chemistry [82]. A second layer of glycosyl residues (maltose or lactose) containing thioctic acid was covalently linked on supported AuNPs, affording the fiber-type “Sugar Chips” (Figure 6). By means of this sugar chips the carbohydrate−protein interactions have been analyzed and quantified in a very small volume with results confirmed by the conventional LSPR biosensors.

Gibson et al. [66] designed 40 nm AuNPs capped with glyco-PEG ligands to discriminate different strains of *E. coli*, on the base of different expression level of the bacterial lectin FimH with a colorimetric assay. A simple and fast bioassay has been developed by Lee et al. [83] to recognize cholera toxin (CT), a protein secreted by the *Vibrio cholerae* bacterium which is responsible for cholera disease. A thiol-modified β-galactose derivative was used to coat 10 nm AuNPs and mixed with amino-terminated quantum dots (CdTe, QD). As a consequence of the strong hydrogen bonds among QD amines and hydroxy groups of galactose, a complex of QD-AuNPs occurred as confirmed by using fluorescence resonance energy transfer (FRET). In the presence of the analyte (i.e., cholera toxin), galactose-AuNPs recognize the protein, avoiding the formation of QD complex and, consequently recovering the fluorescence. This method, although based on a monosaccharide, showed the impressive improvement obtained for using multivalent glyco-nanosystems.

Quick serological detection of cancer biomarkers is one of the main aim in early cancer diagnosis, and GAuNPs seem to be fundamental in the development of new, cheap and easy tests. Tian et al. [28] described the design of mannosylated gold nanoparticles which create a supramolecular glycoprobe in the presence of a specific mannose-selective *Lens culinaria* lectin (LcA). This lectin is particularly interesting because it can have a strong affinity with the core-fucosylated glycoform of α-fetoprotein (AFP-L3), a recognized biomarker for the diagnosis of hepatocellular carcinoma. When the glycoprobe interact with a microplate displaying AFP-L3, LcA strongly bind to AFP-L3 releasing from the supramolecular glycoprobe GAuNPs in a concentration which can be measured via UV–vis spectroscopy. Chiodo et al. [84] gave their contribution in the development of early stage cancer or pathogen diagnosis designing an ELISA approach to detect anti-carbohydrate antibodies. The innovative procedure exploited the use of very small AuNPs (2 nm) coated with tetrasaccharide epitopes of HIV gp120 or tetrasaccharide epitopes of *Streptococcus pneumoniae* Pn14PS. The GAuNPs so obtained were directly coated on commercial ELISA plates and anti-carbohydrate antibodies were detected in the nanomolar range both using purified anti-HIV human monoclonal antibodies or serum from immunized mice against *S. pneumoniae*.

#### Detection of carbohydrate–influenza virus interactions

The SERS methodology was extended to the determination of the binding selectivity for human and avian influenza (H1N1 and H7N9), by using modified glycans (lactose, α(2,3)Neu5AcLacNAc and α(2,6)Neu5AcLacNAc) to introduce the active moieties on the gold monolayer. The SERS spectra registered with changes in the 1400–1600 and 1000–1200 cm^−1^ bands were indicative of the successful binding [85]. In this field, a naturally occurring sialylglycopeptide extracted from egg yolks was converted into a thiol-terminated molecule and grafted on AuNPs, to develop a gold-based sensor for influenza virus detection, using both UV–vis spectroscopy and dynamic light scattering. Moreover, a selective detection of the influenza virus can be accomplished. Viral hemagglutinin (HA) protein, in fact, can bind selectively sialic acid residues, but the human influenza virus recognizes the sialic acid α(2,6)galactose sequence while the avian virus recognizes sialic acid α(2,3)galactose chains [85]. GAuNPs functionalized with the exact sequence have been able to selectively recognize the influenza virus strain by means of a simple colorimetric assay, observing the hemagglutinin-induced aggregation of AuNPs [44–45]. Moreover, to improve the sialic residue display on metal surfaces, Russel and co-workers [45] employed trivalent α-thio-linked sialic residues as ligands of AuNPs, confirming the improving ability in binding selectively the human influenza strain.

#### Detection of carbohydrate–peptide hormone interactions

Dextran-coated AuNPs have been employed to investigate the binding properties of the glucose moiety displayed on a metal surface with insulin, a peptide hormone [86]. By means of a UV–vis spectroscopy assay, the authors confirmed the successful use of dextran-coated AuNPs to selectively bind low concentrations of insulin (at 1 pM), even in serum samples, and discovered that the insulin B chain is mainly responsible for the binding with glucose moiety AuNPs.

### Glyco-gold nanoparticles as drug carriers and contrast agents

Drug nanocarriers play a fundamental role in the development of nano- and personalized medicine. The main advantages resulted from the protection of the drug from degradation or inactivation in vivo, the opportunity to control the drug release and reduce the systemic toxicity and the exciting chance to selectively target the damaged tissue. Despite the most common carriers are based on nano- and microparticles (i.e., albumin, poly(lactic-*co*-glycolic acid)-PLGA NPs, liposomes), many examples in literature report on the development of gold-based glyco nanoparticles as efficient system to delivery payloads. In this contest, AuNPs modified with lactose moieties and β-cyclodextrins have been reported by Vargas-Berenguel et al. [87]. The authors showed that GAuNPs were still able to recognize specific lectin (peanut agglutinin, PNA) and human galectin-3 (Gal-3), exploiting multiple lactose residues displayed on gold surface. Moreover, due to the presence of the β-cyclodextrin cavity, these nanostructures worked as site-specific delivery systems, hosting methotrexate (MTX), an anticancer drug.

**Figure 7:**
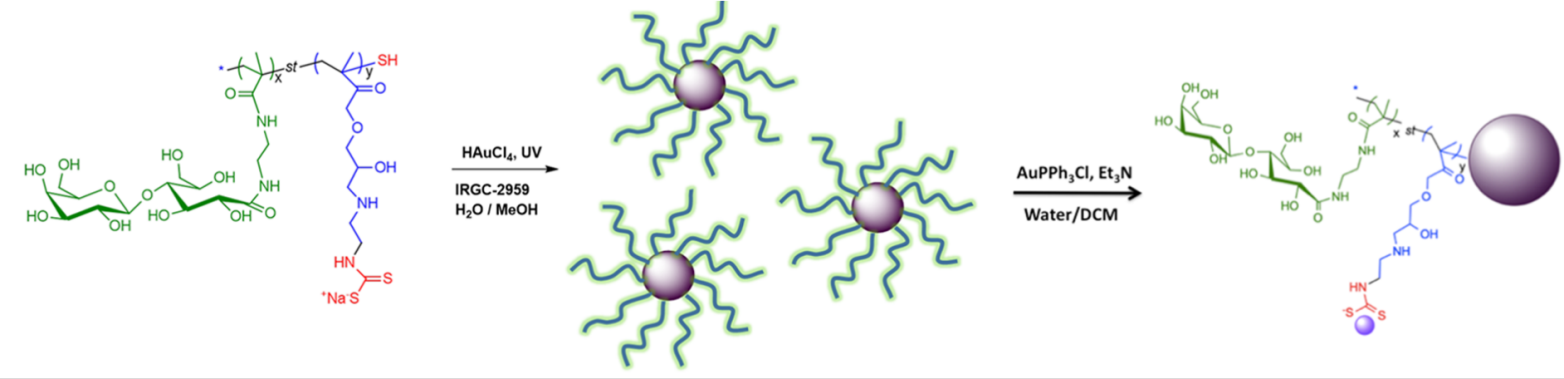
Design of GAuNPs, displaying both sugar residues and the anticancer Au(I)PPh_3_. Reprinted with permission from [88]. Copyright 2014 American Chemical Society.

The anticancer therapy based on platinum compounds, such as cisplatin, carboplatin and oxaliplatin, has many positive effects on the regression of cancer cell proliferation. Unfortunately these compounds are low tolerated by the organisms, therefore new metal-based anticancer drugs need to be developed. Recently, it has been reported the interesting synthesis of AuNPs coated with glyco-polymers and functionalized with gold(I) triphenylphosphine (Figure 7) [88]. This work showed the potentiality of these structures as novel cancer therapeutic drugs. The glucose and galactose moieties multi-displayed on gold surface acted as target for asialoglycoprotein receptors, over-expressed on liver cancer cells, delivering selectively the anticancer drug Au(I).

In search of novel antimicrobial antigens, chitosan-streptomycin GAuNPs (CA NPs), i.e., carbohydrate-antibiotic conjugates, were prepared and resulted able to overcome antibiotic resistance of microbial biofilms, since CA NPs render streptomycin more accessible to biofilms, thereby more available to interact with biofilm bacteria [89]. Similarly, a novel recyclable *E.coli*-specific killing GAuNP nanocomposite, carrying either a targeting agent towards *E. coli* pili and an antibacterial polymer, resulted able to bind *E. coli* specifically and to kill the attached bacteria, by enhancing the local concentration of biocidal agent on the bacterial surface [90].

GAuNPs find interesting application also in the treatment of HIV infection. β-D-Glucose coated AuNPs have been decorated with the antiviral drugs abacavir (ABC) and lamivudine (3TC) and used as delivery systems. These co-drug structures were able to release the drugs in acidic conditions to inhibit viral replication in cellular assays [91].

GAuNPs represent a promising candidate for developing theranostic agents, able to carry drugs to a selected tissue and to act as a sensitive probe. In fact, the metallic core of GAuNPs has been exploited to design and develop promising early detecting agents of cancer or inflammation. In this contest, Andresen and co-workers produced a nanogel injectable in humans to detect cancer cells. The nanogel was composed by sucrose acetate isobutyltyrate (SAIB) and poly(*N*-isopropylacrylamide) (PNIPAM)-coated AuNPs, compatible with SAIB matrix. The nanogel had good opacity for 2D X-ray visualization and was tested in vivo in immunocompetent healthy NMRI-mice and in humans [92]. The gold high X-ray absorption and a carbohydrate coating have been also exploited by Polito and co-workers to produce functional X-ray contrast agents based on glucosamine-coated AuNPs [22]. By micro-CT analysis performed on lung injured mice, the authors demonstrated that GAuNPs selectively targeted overexpressed glucose receptors (GLUT1) and accumulated in the damaged lung, while PEGylated AuNPs did not show any relevant accumulation in the inflammation site. Moreover, another study on the synthesis of glucose-functionalized AuNPs demonstrated that GLUT1-targeting GAuNPs are well compatible with blood and do not significantly interact with ovine red blood cells [93]. In the last years, numerous compounds containing Gd^3+^ have been developed and used as magnetic resonance imaging (MRI) contrast agents. Recently, gold based nanosystems containing Gd^3+^ were developed. Arus et al. described the synthesis of water dispersible GAuNPs decorated with Gd complexes [94]. The glyco-shell conferred to the nanotracer a targeting properties while Gd^3+^ acted as MRI contrast agent. The authors settled an ex vivo experiment which let them to use small amounts of contrast agent (5 nmol/animal), avoiding toxicology and in vivo test. Moreover, GAuNPs showed their extreme versatility in a very interesting paper reported by Penadés and collaborators [11]. They design GAuNPs able to pass the brain blood barrier (BBB) of healthy mice and give images of intact brain. AuNPs were decorated in a controlled ratio with glucose, neuropeptides and a chelator of gallium-68, a sensitive positron emission tomography (PET) tracer, affording the quantification of BBB permeability in healthy small animals.

### Glyco-gold nanoparticles in immunology

The development of nanoparticle-based strategies for the modulation of the immunosystem represents one of the biological and biomedical applications of nanomaterials. Nanoparticle technologies offer the possibility of new approaches to tune specific immune responses for prevention or treatment of diseases. These approaches are inspired by the availability of novel nanomaterials with specific capabilities [95]. AuNPs have attracted great attention in this field due to their unique characteristics of biocompatibility and easy fabrication, already outlined previously. In the last five years, significant examples on the use of carbohydrate-coated GAuNPs in the field of immunology have been reported, addressing different possibilities of intervention on the immune system.

#### GAuNPs and activation of the immune system via cellular targeting

Glycan-conjugated globular shaped nanoparticles have a higher surface area to volume ratio in comparison to other glycoclusters, i.e., glycolipid micelles and glycoproteins, which allows an ordered, controlled and stable tridimensional presentation of the ligands, providing higher ligand binding capacity [6,96]. For this reason, they represent an ideal scaffold to study the activation of the immune system in response to pathogens mediated by lectins, like DC-SIGN. Mammalian cell surface lectins, expressed on the surface of dendritic cells (DC) and macrophages, recognize in a multivalent way a vast array of glycans on the exterior of pathogens. Natural carbohydrate ligands include high-mannose N-glycans, such as those found on the HIV gp120 protein, and Lewis-type glycans. Ligand recognition often initiates events which are able to trigger specific signaling and result in a robust immune activation in humans. For example, galactofuranose (Gal*f*) functionalized AuNPs were found able to elicit a pro-inflammatory response in dendritic cells via interaction with the lectin DC-SIGN, as indicated by the up-regulation of several maturation markers and increased secretion of pro-inflammatory cytokines interleukin 6 (IL-6) and tumor necrosis factor α (TNF-α) [97]. These data indicate that a multivalent system of Gal*f*, a five-membered form of galactose found in nonmammalian pathogenic species, i.e., the fungus *Aspergillus*, is able to modulate the innate immune response via dendritic cells, suggesting Gal*f*-GNPs as versatile tools in functional studies to address the role of Gal*f* in host-pathogen interaction.

DC-SIGN targeting through GAuNPs has also been exploited differently in a study on GAuNPs functionalized with α-fucosylamide, an efficacious synthetic DC-SIGN ligand, analogue of the natural fucose-containing Lewis^x^ trisaccharide [98]. This paper shows that GAuNPs bearing 50% of fucosylamide are able to compete with the natural gp120 ligand on DC-SIGN expressing cells, and to promote DC-SIGN internalization. On the contrary, the GAuNPs lack any immunomodulatory activity, and do not induce DC maturation or trigger cytokine responses. Thus, in this example, GAuNPs, can be considered as vehicles, neutral carriers, to target antigens to DCs via lectins, a well-known strategy explored in immunotherapy.

#### GAuNPs in vaccine development

AuNPs engineering is offering significant contribution to immunology also in vaccine development. The repetitive antigen display is the key point related to this nanotechnology-based approach, which aim to trigger the production of specific and functional antibodies that prevent initial infection limiting pathogen/viral dissemination. Recent publications suggest that many different aspects are becoming clear and have to be underlined.

The design of anti HIV-1 vaccines depends on the identification of sugar epitopes of the surface envelope glycoprotein of HIV-1, capable of eliciting a protective response. GAuNPs coated with high-mannose type oligosaccharide of HIV-1 gp120, were prepared as glycoconjugate systems able to mimic the natural presentation of gp-120 high-mannose glycans. It was demonstrated that they were able to inhibit DC-SIGN-mediated HIV-1 infections and to interfere with the antibody/gp120 binding process. Most of these systems proved able to elicit the production of carbohydrate-specific antibodies in animals, though the generated IgGs turned out to be unable to neutralize the virus. More recently, the Man_9_GlcNAc_2_ was identified as a conserved motif on the HIV surface: this result stemmed from the observation that partial structures of this glycan could be recognized by broadly neutralizing antibodies, found in infected patients. These data suggested the use of these self-glycans for the design of effective vaccine and inspired the preparation of new generation GAuNPs, coated with synthetic partial structures of Man_9_ multimerized on the same GAuNP, which provided better binding to the anti-HIV antibody 2G12 compared to GAuNPs carrying only one individual oligomannoside [99]. This result, based on the assembly of “antennas” on the NP surface, shows that the cluster presentation of the gp120 high-mannose type glycan on GAuNPs provides different binding modes and hence more opportunities to mimic the multivalent interaction between the antibody and the saccharide. Finally, these results represent a solid support for the design of new and more effective nanoparticles based systems.

A different approach towards the development of a synthetic HIV vaccine candidate based on GAuNPs, exploit the glyco-nanosystem as a tool which modulate and control the biologically active conformation of a significant epitope [100]. In this respect, negatively charged GAuNPs were conjugated to the third variable region (V3 peptide) of the HIV-1 gp120. The V3 peptide is a major immunogenic domain of HIV-1, which can be exploited as a candidate for anti-HIV vaccine development, given that the immunologically active conformation is conserved. The peptide on the nanoparticle showed increased stability towards degradation as compared to the free peptide. Moreover, V3β-GAuNPs elicit antibodies in rabbits that recognize a recombinant gp120, even in the absence of highly immunogenic proteins.

Another example on the importance of the way in which glycoconjugates are presented through NPs, regards the design and construction of GAuNPs decorated with mucin-related glycans for cancer immunotherapy [57]. A monomolecular sugar coating do not represent well the structure of mucin glycoprotein on the surface of cancer cells, which are characterized by a dense presentation of glycans attached to a protein backbone. So, multicopy–multivalent polymeric versions of the tumor-associated α-GalNAc (Tn) antigen, obtained through RAFT polymerization, were prepared and used to prepare GAuNPs with a surface that mimics much more closely the surface of cancer cells. These systems were able to generate a significant immune response in vivo, with the generation of detectable levels of antibodies specific for naturally occurring mucin glycans, without the need for a immunogenic protein component.

In a different example, Barchi and co-workers focused on the Thomsen Friedenreich tumor-associated antigen (TFag) and synthesized TFag-amino acid-coated AuNPs by means of a thiol-polyethylene glycol spacer. Among the synthesized GAuNPs, TFag-Thr-AuNPs showed, in vitro, an increased cytotoxic activity toward lymphoma cells, resulting active at a very low micromolar range [101]. With the aim to activate the human immune system against self-tumor cells, Schlecht and co-workers designed the synthesis of AuNPs functionalized with Mucin1(MUC1)-glycopeptide antigens [102]. Mucins are a family of glycosylated proteins with a high molecular weight, produced by epithelial tissues. The most studied is the membrane-bound glycoprotein MUC1, a glycoprotein with extensive O-linked glycosylation in its extracellular domain. The authors demonstrated that the multivalent presentation of MUC1-glycopeptide antigens were able to stimulate the immune system, suggesting GAuNPs are interesting platforms for developing new immunotherapeutics.

The potential of AuNPs as antigen carriers for the development of synthetic vaccines is still being investigated in the context of bacterial infections. NPs are indeed promising vector systems to explore vaccination against infectious diseases.

Recently, a study on the identification of an optimal vaccine formulation to protect against listeriosis, proposed the use of GAuNPs loaded with glucose and the LLO_91-98_ *Listeria* peptide antigen as a system to target DCs, since *Listeria* protection is largely dependent on T-cells activation [103]. The presence of glucose ensures a biocompatible and water dispersible highly efficacious DC targeting system. DC loaded in vitro with GAuNPs-LLO, co-formulated with an adjuvant, provided better protection against listeriosis than DC loaded in vitro using free LLO peptide, and induced LLO-specific T-cell immunity and protection against *Listeria*. The system was effective in mice immunization either using a DC vaccine or a standard immunization approach. The data were followed by a study on pregnant mice vaccination which demonstrated the ability of this AuNP based vaccine system to protect neonates born to vaccinated mothers from the bacteria [104].

In another study, the impact of multivalency on the ability of GAuNPs to induce immune cell responses in vitro was studied using highly pure NPs coated with non-immunoactive mono- and disaccharides related to the capsular polysaccharide of serogroup A of Neisseria meningitides [53]. The results showed that the systems were able to activate the antigen-presenting cells, to induce the releasing of effector functions of the same cells and to stimulate T cell proliferation, but highlighted that immunoactivity is strongly dependent on the size of the nanoparticle (larger NPs, 5 nm, performed better than smaller ones, 2 nm) and on the length of the saccharide fragment. There is growing evidence that the shape and size of the AuNPs affect immunological responses in vitro and in vivo [105].

In another recent study on the development of synthetic nanosystems as potential carbohydrate-based vaccine, GAuNPs were loaded with synthetic oligosaccharide fragments corresponding to the repeating units of *S. pneumoniae* (Pn) CPS type 19F and 14 [106]. This new approach has explored the effect of the presence of two different saccharide epitopes from diverse bacterial serotypes simultaneously displayed onto the NP surface on the immunological response. Mice immunization showed that the concomitant presence of Pn14 and Pn19F repeating unit fragments on the same NP critically enhance the titers of specific IgG antibodies towards type 14 polysaccharide compared with GAuNP exclusively displaying Pn14.

The GAuNP technology has also been applied in the regulation of important genes of the apoptotic pathway. New GAuNP systems displaying cMyc targeting siRNA resulted capable of inducing apoptosis via hyperactivation of cell death receptors and caspase pathways [107].

## Conclusion

Glyco-gold nanoparticles represent one of the most versatile, exciting and promising hybrid nanosystems. Combining the vast properties of the noble metal at its nano-size with the fundamental biological role covered by carbohydrates, GAuNPs hold great potentiality in bio-nanomedicine. In this review, we have attempted to summarize the last results obtained in this field, anticipating the numerous applications with a detailed description and implication of each part of the hybrid system. A careful GAuNP design must take into consideration all the parameters which characterize the final glyco-nanoparticle. Well planned GAuNPs offer a unique chance to researchers to mimic the nature’s glycocalix and to explore the world of weak carbohydrate interactions, opening the way to innovative diagnostic tools or new therapeutic or theranostic agents.
